# Chemokine CXCL1 mediated neutrophil recruitment: Role of glycosaminoglycan interactions

**DOI:** 10.1038/srep33123

**Published:** 2016-09-14

**Authors:** Kirti V. Sawant, Krishna Mohan Poluri, Amit K. Dutta, Krishna Mohan Sepuru, Anna Troshkina, Roberto P. Garofalo, Krishna Rajarathnam

**Affiliations:** 1 Department of Biochemistry and Molecular Biology, The University of Texas Medical Branch, Galveston, TX, USA; 2 Department of Microbiology and Immunology, The University of Texas Medical Branch, Galveston, TX, USA; 3 Department of Pediatrics, The University of Texas Medical Branch, Galveston, TX, USA; 4 Sealy Center for Structural Biology and Molecular Biophysics, The University of Texas Medical Branch, Galveston, TX, USA

## Abstract

The chemokine CXCL1/MGSA plays a pivotal role in the host immune response by recruiting and activating neutrophils for microbial killing at the tissue site. CXCL1 exists reversibly as monomers and dimers, and mediates its function by binding glycosaminoglycans (GAG) and CXCR2 receptor. We recently showed that both monomers and dimers are potent CXCR2 agonists, the dimer is the high-affinity GAG ligand, lysine and arginine residues located in two non-overlapping domains mediate GAG interactions, and there is extensive overlap between GAG and receptor-binding domains. To understand how these structural properties influence *in vivo* function, we characterized peritoneal neutrophil recruitment of a trapped monomer and trapped dimer and a panel of WT lysine/arginine to alanine mutants. Monomers and dimers were active, but WT was more active indicating synergistic interactions promote recruitment. Mutants from both domains showed reduced GAG heparin binding affinities and reduced neutrophil recruitment, providing compelling evidence that both GAG-binding domains mediate *in vivo* trafficking. Further, mutant of a residue that is involved in both GAG binding and receptor signaling showed the highest reduction in recruitment. We conclude that GAG interactions and receptor activity of CXCL1 monomers and dimers are fine-tuned to regulate neutrophil trafficking for successful resolution of tissue injury.

Circulating neutrophils are rapidly recruited in response to microbial infections and form the first line in host defense. Impaired recruitment and/or impaired activation could result in runaway infection leading to sepsis, whereas uncontrolled recruitment and/or sustained activation could result in collateral tissue damage and disease^1^,^2^,^3^,^4^. Clinical studies and animal models have shown that the chemokine CXCL1 plays dual roles in the host immune response by recruiting and activating neutrophils to combat infection. First, it acts as a directional cue in routing peripheral neutrophils to the site of infection. Second, it activates the release of proteases and reactive oxygen species (ROS) for microbial killing in the tissue^5^,^6^,^7^,^8^,^9^.

CXCL1 (also known as MGSA and Gro-α in humans and KC in mice) mediates function by signaling via CXCR2 on neutrophils and by binding to glycosaminoglycans (GAG) on endothelial and epithelial cells and the extracellular matrix (ECM). GAGs such as heparan sulfate are a family of linear polysaccharides covalently attached to core proteins called proteoglycans, and are present on both the luminal and abluminal sides of the endothelium and epithelium and as non-covalent complexes with proteins such as collagen and laminin in the ECM^10^,^11^. In principle, GAG interactions can result in soluble chemotactic and tissue-bound haptotactic gradients, and the nature of these gradients can vary in the vasculature, across the endothelium, and across the epithelium^12^,^13^,^14^. ECM GAGs can also be cleaved by proteases during the recruitment process, and soluble GAG-bound chemokine gradients have been shown to regulate neutrophil migration^15^. GAG interactions can also regulate recruitment by increasing chemokines’ lifetime by preventing its proteolysis and from being washed away by blood flow in the vasculature^16^. All of these processes collectively control the flux, duration, and kinetics of circulating neutrophil egress to the tissue for successful resolution of inflammation.

CXCL1 levels are negligible under basal conditions but increase significantly during active infection. CXCL1 exists as monomers and dimers (dimer dissociation constant ~10 μM), and therefore one or both forms could mediate trafficking. We recently showed that both monomers and dimers are agonists for the CXCR2 receptor and that the dimer binds GAG with higher affinity^17^,^18^. We also showed that basic residues located in two distinct domains on opposite faces of the protein orchestrate GAG interactions, and some of the GAG-binding residues are also located in the receptor-binding domain indicating that GAG-bound chemokine cannot activate the receptor^18^.

In this study, we characterized how CXCL1’s structural properties influence *in vivo* function, by characterizing the peritoneal neutrophil recruitment of the WT (that can exist as monomers and dimers), a trapped non-associating monomer, a trapped non-dissociating dimer, and alanine (Ala) mutants of lysines and arginines from both GAG-binding domains on the WT background. On the basis of our current and previous studies on the related chemokine CXCL8, we propose that GAG interactions and receptor activity of the monomer and dimer collectively determine *in vivo* gradients in a chemokine-specific manner to direct neutrophils to the target site.

## Results

### Recruitment profiles of CXCL1 variants

**Figure 1:**
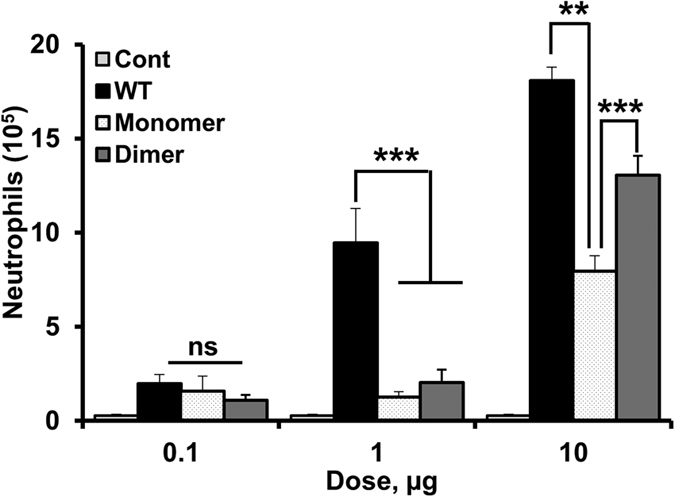
Neutrophil recruitment profiles of CXCL1 wild type, monomer and dimer at different doses. BALB/c mice were injected intraperitoneally with different doses (0.1, 1 and 10 μg) of the CXCL1 variants. Peritoneal cells were harvested 6 h post-injection and total cells were counted along with differential counting to measure neutrophil levels. The results are expressed as means ± standard error from two independent experiments using 4 animals/group. The levels of neutrophils for both monomer and dimer are compared to WT at each dose and the differences between the variants were compared using ANOVA followed by Tukey’s post hoc analysis. **p < 0.01, ***p < 0.001.

We characterized the peritoneal neutrophil recruitment of WT CXCL1 and of the trapped monomer and dimer at different doses with PBS as the control (Fig. 1). The trapped monomer cannot dimerize and the trapped dimer cannot dissociate at any concentration or condition. Accordingly, the recruitment can be unambiguously attributed to the monomer or dimer. We have shown previously that the structures of the trapped variants are essentially similar to the native chemokine^17^,^19^.

We observed that the recruitment profiles vary, and in particular, the monomer and dimer were minimally active at low doses, and the WT was more active than the monomer and dimer at all doses. CXCL1-mediated trafficking involves GAG interactions and CXCR2 activation. We have previously shown that both monomer and dimer are potent agonists for receptor activation and that the dimer is the high affinity GAG ligand^17^,^20^. Therefore, we attribute the low recruitment activity of the dimer at lower doses to it predominantly existing in the GAG-bound form, and of the monomer to sub-optimal gradients due to its low GAG affinity. The higher activity of the WT, which is especially evident at the 1 μg data, must be due to its ability to exist as monomers and dimers, which allow optimal gradients for efficient recruitment to the tissue.

**Figure 2:**
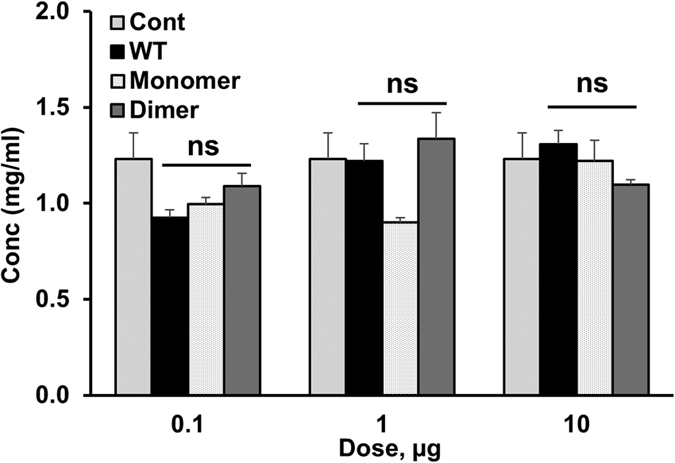
Protein levels in peritoneal fluid. Peritoneal fluid supernatants from mice were estimated using the BCA assay kit (Pierce). Each data set is representative of two experiments using 4 animals/group. Protein levels for each variant were compared to the PBS control using ANOVA followed by Tukey’s post hoc analysis (p < 0.05). (ns - not significant).

We also measured protein levels in the peritoneum as an indicator of tissue damage. Robust recruitment and/or premature neutrophil activation could damage the integrity of the epithelium resulting in the leakage of plasma proteins into the peritoneum^21^,^22^. We observed that the protein levels of the WT and monomer and dimer variants at all doses were similar, and also no different from the PBS control (Fig. 2). In particular, that robust recruitment observed at the 10 µg dose, which is comparable to that seen in disease models, does not cause any tissue damage is quite striking. These observations suggest that the process of chemokine-mediated recruitment itself is highly regulated, and tissue damage observed during infection can be attributed to premature or uncontrolled neutrophil activation.

### Design of CXCL1 GAG mutants

**Figure 3:**
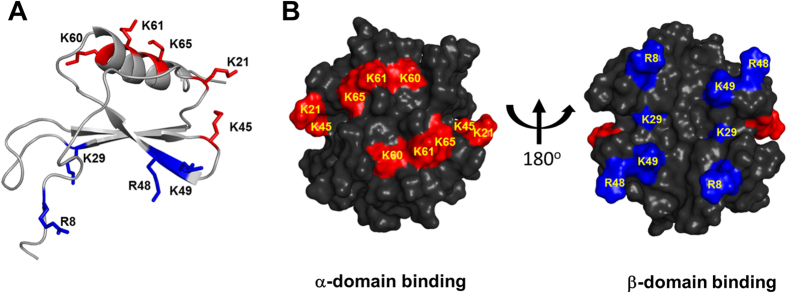
GAG binding domains in CXCL1. **(A**) Ribbon representation of the CXCL1 monomer. α-domain and β-domain GAG-binding residues are highlighted in red and blue, respectively. (**B**) Surface representation of the CXCL1 dimer showing the GAG-binding residues of the α-domain and β-domain are in red and blue, respectively.

We recently showed that the GAG-binding residues are located in two non-overlapping domains and that the chemokine-GAG interface is extensive^18^ (Fig. 3). Residues Lys21 from the N-loop, Lys45 from the 40 s turn, and Lys60, Lys61, and Lys65 from the C-helix from both monomers of the dimer, constitute one domain that we define as the α-domain. Residues Arg8 from the N-terminus, Lys29 from the 1^st^ β-strand, Lys48 from the 40 s turn, and Lys49 from the 3^rd^ β-strand from both monomers of the dimer, constitute the other domain that we define as the β-domain. In principle, role of α- and β-domains and of the individual residues in each domain could be the same or different. For instance, one of the domains and/or specific residues from each domain could play a more prominent role and differentially regulate function. In order to explicitly address the potential roles for GAG interactions, we individually mutated these basic residues to alanine (Ala) and characterized their GAG binding affinities and neutrophil recruitment activities.

### GAG binding affinities of CXCL1 mutants

The binding affinities of the CXCL1 mutants were measured for GAG heparin using surface plasmon resonance (SPR). Heparin is commonly used for structural and biophysical studies for the reason it is commercially available, and has been shown to be useful for determining relative affinities and for identifying GAG-binding residues. The most common GAG on cell surfaces is heparan sulfate that has a modular structure of sulfated (NS) and acetylated (NA) domains whereas heparin is more uniformly sulfated. It has been shown that two NS domains separated by an NA domain in heparan sulfate binds chemokine CXCL8 dimer, and also that native chemokines dimerize on binding to cell surface GAGs^23^,^24^.

**Figure 4:**
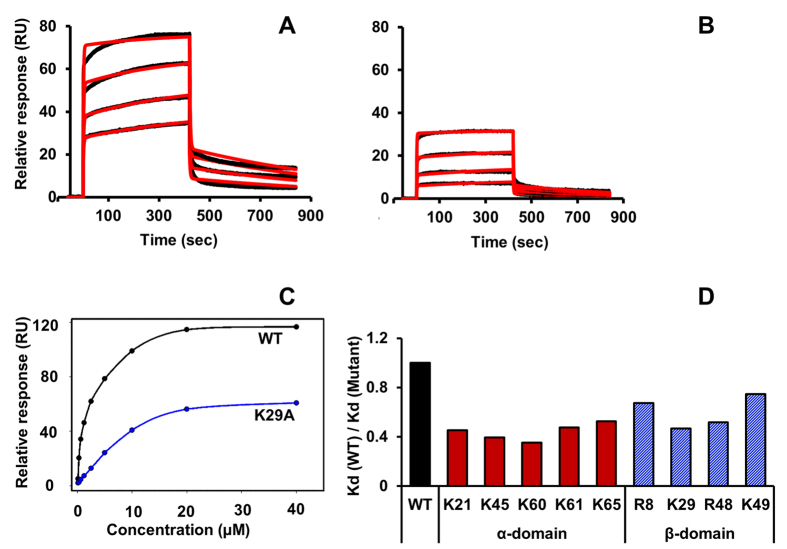
Binding affinities of CXCL1 GAG mutants. SPR sensorgrams for (**A**) CXCL1 WT and (**B**) K29A mutant passed over immobilized heparin on a BIAcore SA chip. The curves represent the maximum response signal (RU) minus the reference signal (no GAG). Kinetic fits for the raw data are shown in red lines. (**C**) Steady state K_d_ analysis. Maximum response units were plotted for each concentration of CXCL1 WT and K29A mutant. (**D**) Relative binding affinities of the α-domain and β-domain GAG mutants compared to the WT.

Binding affinities (K_d_) were calculated from surface plasmon resonance (SPR) measurements^25^. Binding profiles for the WT and K29A mutant and a summary of the binding affinities are shown (Fig. 4). The binding affinities for the WT and dimer were similar ~2 ± 1 μM suggesting that the WT binds heparin as a dimer. The binding constants were calculated using the steady state model. Data could be fitted for all mutants with low χ^2^ values (between 0.2 and 2.5), and the calculated binding constants show that the mutants bind heparin with 2 to 3 fold lower affinity (Fig. 4D). We also fitted the SPR data to Langmuir model, where the binding constant was calculated from the kinetic on and off constants. These data show that the mutants bind with 3 to 8-fold lower affinity, with χ^2^ values between 2.7 and 8.8. The calculated and experimental fits for the WT and K29A mutant using the Langmuir model are shown in panels A and B, respectively.

The reported binding constants are apparent binding constants and have contributions from both monomer-dimer equilibrium and binding affinities of the monomer and dimer. Binding affinities of the monomer and dimer will vary, and we have shown previously using NMR spectroscopy that the dimer is the high-affinity ligand^18^. Mutations do not influence the monomer-dimer equilibrium and contribution from the monomer to overall binding will be less due to its lower binding affinity. Therefore, any differences in binding affinity essentially arise from reduced binding of the mutant dimer due to loss of H-bonding/electrostatic interaction mediated by that basic residue. The *in vivo* recruitment activity of the mutants is lower and correlates with the reduced heparin binding affinities.

### Receptor activity of CXCL1 GAG mutants

**Figure 5:**
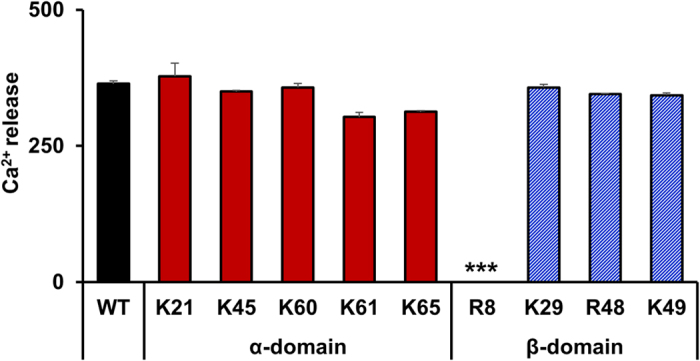
Receptor activity of GAG mutants. Receptor activity of the CXCL1 mutants was characterized by measuring calcium release in CXCR2 transfected HL60 cells using FLIPR assay. 10 nM of CXCL1 mutants were added to the cells in quadruplicate and raw peak fluorescence was obtained as relative fluorescence units (RFU). Data shown are mean ± SEM of the four measurements, and the graph is a representative of three independent experiments. The calcium release activity for each mutant was compared with the WT using ANOVA followed by Tukey’s post hoc analysis. ***p < 0.001.

CXCR2 receptor activation can be characterized by downstream functions like calcium release^17^,^26^. We therefore characterized calcium release as a read-out for receptor function (Fig. 5). Our data show all the mutants, except R8A, are as active as the WT indicating that the altered recruitment is essentially due to altered GAG interactions. The R8A mutant showed no activity and was also highly impaired in the recruitment studies.

### Peritoneal recruitment of CXCL1 mutants

**Figure 6:**
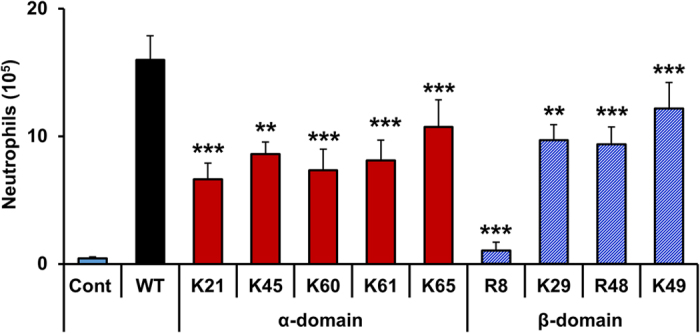
Neutrophil recruitment of CXCL1 GAG mutants in the peritoneum. Mice were injected intraperitoneally with 10 μg of each CXCL1 mutant and cells were harvested at 6 h post-injection. Total cells were counted and a differential cell count was done. The results are expressed as means ± standard error from two independent experiments using 4 animals/group. The neutrophil levels for the mutants are compared to the WT using ANOVA followed by Tukey’s post hoc analysis. **p < 0.01, ***p < 0.001.

Peritoneal neutrophil recruitment of the GAG mutants was characterized at the 10 μg dose because the recruitment levels seen at this dose are comparable to those seen in disease models^2^,^8^. All mutants were less active providing unambiguous evidence that both α-domain and β-domain GAG interactions mediate *in vivo* recruitment (Fig. 6). Highly impaired recruitment of N-terminal Arg8 mutant must be due to its dual role of binding to GAG and activating the CXCR2 receptor. Previous studies have established that chemokine N-terminal ELR residues are crucial for receptor activation^27^. β-domain N-terminal Arg8 is part of the highly conserved ELR triad, and the Arg8 mutant was also highly impaired in alveolar recruitment^28^.

## Discussion

Chemokines were first identified as critical regulators of immunity about thirty years ago, and it has now become increasingly clear that their function is intimately coupled to not only their receptor activation, but also to their dimerization/oligomerization and GAG binding properties^18^,^25^,^29^,^30^,^31^,^32^,^33^. However, the molecular basis of how these properties orchestrate *in vivo* function is not well understood. CXCL1 belongs to a subset of seven chemokines that are characterized by the highly conserved N-terminal ELR triad and are agonists for the CXCR2 receptor. In this study, we show that both CXCL1 monomer and dimer are competent in mediating *in vivo* neutrophil recruitment, but the WT is more active. We attribute this to WT’s ability to reversibly exist as monomers and dimers. Previous studies using zebra fish CXCL8 mutants that fail to bind proteoglycans *in situ* have shown GAG interactions not only mediate directional guidance but also restrict neutrophil motility suggesting that these distinct functions are most likely mediated via differential activities of the monomers and dimers^34^. We also show that residues located at two distinct domains mediate *in vivo* GAG interactions, and that interactions from both domains are important as mutating even one of the GAG-binding residues impacts recruitment.

Comparison of the recruitment properties of CXCL1 and related chemokine CXCL8 show similarities and differences^32^. For both chemokines, WT is more active than the monomer or dimer, but the recruitment profiles vary for the WT and monomer but not for the dimer. For both chemokines, the dimer binds GAG with higher affinity but their GAG interactions are quite different. CXCL8 binds only residues of the α-domain, as well as some peripheral residues in the same face of the protein. Conversely, in addition to binding to the α-domain, CXCL1 binds another domain (defined as the β-domain) on the opposite face of the protein^18^,^33^.

Migration of circulating neutrophils to the injury site is a multi-step process, and involves initial arrest in the vasculature, and subsequent migration across the endothelium, extracellular matrix, and epithelium. Chemokines mediate recruitment at every step of the way by forming gradients that involve GAG interactions^12^,^13^,^14^. Structural studies have shown that the N-loop and N-terminal residues of CXCL1 in the GAG-bound form are not accessible for receptor binding, indicating that only soluble and not GAG-bound gradients mediate neutrophil trafficking^18^. Structural studies of CXCL8 in the GAG-bound form have also shown that the N-loop residues are occluded and not accessible for receptor binding suggesting soluble chemotactic gradients mediate trafficking^33^. However gradient characteristics such as steepness at different locations along the migration route most likely vary between the two chemokines, which could explain the differences in their neutrophil trafficking profiles. We propose that crosstalk between GAG and receptor interactions of monomer and dimer dictate gradients at different locations, and that differences in gradients determine chemokine-specific differences in neutrophil trafficking.

**Figure 7:**
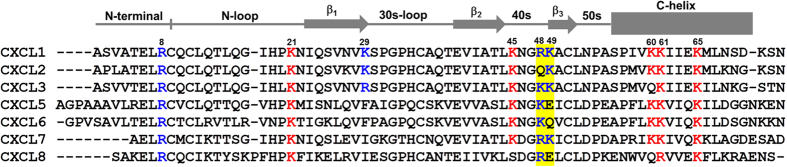
Sequences of ELR chemokines. Basic residues of α-domain and β-domain in CXCL1 and other ELR chemokines are highlighted in red and blue, respectively. The BB motif in CXCL1 and the corresponding residues in other chemokines are shaded in yellow.

Our previous studies have shown that the structural basis underlying GAG interactions of CXCL1 and CXCL8 are distinctly different. Comparison of the ELR chemokine sequences reveal similarities and sequence-specific differences in the distribution of the lysine and arginine residues (Fig. 7). For instance, a ‘BB’ motif with one residue from the 40 s turn and the other from the 3^rd^ β-strand, are conserved in CXCL1, CXCL3, and CXCL7, whereas other members including CXCL8 have only one of the two basic residues. On the other hand, N-terminal arginine is absolutely conserved, which is involved in GAG interactions in CXCL1 but not in CXCL8. These observations suggest that presence or absence of a few basic residues in the context of structure could elicit strikingly different GAG interactions. Our studies on CXCL5 have already shown that its GAG interactions differ from those of CXCL1 and CXCL8. Chemokine receptor activation triggers G-protein and β-arrestin signaling pathways, and both pathways play a role in the migration process. However, related chemokines can have similar potencies and efficacies for some functions but different for others suggesting that receptor function is also highly context dependent^35^. Our current and previous studies collectively indicate that structural and functional properties of ELR chemokines, at first glance, may look deceptively similar, but in reality may be quite different in the context of *in vivo* recruitment. We conclude that the individual chemokines have evolved to maximally exploit GAG and receptor interactions of monomers and dimers to fine-tune their *in vivo* functional response.

## Methods

### Animals

Seven to eight week old female BALB/c mice (Harlan, Houston, TX) were maintained in pathogen-free conditions in the animal research facility (ARC) of UTMB. All mouse experiments were carried out in strict accordance with the Guide for the Care and Use of Laboratory Animals of the National Institutes of Health. The protocol was approved by the Institutional Animal Care and Use Committee of the University of Texas Medical Branch at Galveston (Protocol: 0702005).

### Design and expression of human CXCL1 variants

The trapped CXCL1 dimer was designed by mutating Asn-27 with cysteine, which results in a disulfide across the dimer interface^17^. The trapped monomer was designed by substituting the dimer interface residue Val26 with a non-natural amino acid N-methyl Val, which disrupts H-bonding interactions and also introduces steric bulk at the dimer interface^20^. The trapped monomer was prepared by peptide synthesis (ProImmune, Oxford). The CXCL1 GAG mutants and the trapped dimer were produced as recombinant proteins. The mutant protein genes were generated by PCR amplification using the Quikchange Site-directed mutagenesis kit (Stratagene). The recombinant proteins were expressed as a thioredoxin fusion protein with a His-tag in *E. coli* BL21 (DE3) strain. Transformed *E. coli* BL21 (DE3) cells were grown to an *A*_600_ of 0.6, induced with 0.2 mM isopropyl β-D-thiogalactopyranoside. The cells were grown at 25 °C in the shaker for 12 to 16 hrs before harvesting. The proteins were purified using standard Ni-NTA affinity and reverse-phase high pressure liquid chromatography. The purity and molecular weight of the proteins were confirmed using mass spectrometry and SDS-PAGE.

### CXCR2 receptor activity

Receptor activity of the mutants was measured from changes in intracellular Ca^2+^ levels using the Calcium 6 assay kit (FLIPR No-wash kit, Molecular Devices, Sunnyvale, CA) on a FlexStation III scanning fluorometer. In brief, CXCR2 transfected, differentiated HL60 (dHL60) cells were suspended in HBSS containing 10 mM HEPES and plated in flat-bottomed black microtiter plate (2 × 10^5^ cells/well). The cells were loaded with FLIPR Calcium 6 dye following the manufacturer’s protocol for 1 h. 10 nM of the CXCL1 mutants prepared in HBSS buffer were transferred to the compound plate. Changes in fluorescence were monitored (λ_ex_ 485 nm, λ_em_ 525 nm) every 5 s for 240 s at room temperature after automated addition of mutants to the cells. The agonist response was determined by expressing the maximum change in fluorescence in arbitrary units over baseline.

### Binding affinity measurements

Surface plasmon resonance (SPR) experiments were performed using Biacore T100 (GE Healthcare). 15 kDa heparin (Calbiochem) was biotinylated using the EZ-Link Biotin LC Hydrazide reagent (Pierce) according to a modified version of the manufacturers’ protocol. To create the heparin immobilized chip, a new Sensor Chip SA (Biacore) was conditioned with several injections of 1 M NaCl in 50 mM NaOH, and was then equilibrated with HBS buffer (10 mM HEPES, 300 mM NaCl). The biotinylated heparin was dissolved in the same buffer to a final concentration of 200 ng/ml and injected onto the surface at 10 μL/min to immobilize ~50 RU heparin. The surfaces were conditioned with several injections of 1.5 M NaCl and equilibrated overnight with HBS-EP buffer (10 mM HEPES, 150 mM NaCl, 2 mM EDTA, 0.005% surfactant P20, pH 7.4). The chemokines in HBS-EP buffer were injected onto the immobilized heparin surface at 10 μl/min for 7 mins, and then dissociated by flowing HBS-EP buffer over the surface for 7 mins. Any residual binding was removed by washing with buffer containing 1.5 M NaCl for 7 mins followed by HBS-EP buffer for 4 mins. Protein concentrations from 156 nM to 40 μM were used. Curves were analyzed and fitted using the Biacore T100 evaluation software. All measurements were repeated at least twice, and the calculated binding constants were similar from both the runs.

### Peritoneal neutrophil recruitment

Mice were inoculated intraperitoneally with different doses of CXCL1 variants in 100 μl of DPBS buffer using a 26 gauge needle. The mice were anesthetized 6 h post injection and euthanized by cervical dislocation. The peritoneal cavity was flushed twice with cold PBS to harvest the recruited cells. The cells were centrifuged and the pellet resuspended in PBS for cytospin slides. Slides were fixed, stained with Wright-Giemsa stain, and a differential leukocyte count was performed. Total leukocytes were counted using a hemocytometer after staining with Turk’s solution.

### Statistical analysis

All data are represented as mean ± SEM. The differences between experimental groups were analyzed using one way analysis of variance (ANOVA) followed by Tukey’s post hoc test. Analysis was done using Graph Pad Prism 5 (GraphPad Software). P < 0.05 was considered to be statistically significant.

## Additional Information

**How to cite this article**: Sawant, K. V. *et al*. Chemokine CXCL1 mediated neutrophil recruitment: Role of glycosaminoglycan interactions. *Sci. Rep*. **6**, 33123; doi: 10.1038/srep33123 (2016).
